# Clinical Trials on Cellular Therapy for Children and Adolescents With Cancer: A 15-Year Trend in the United States

**DOI:** 10.7759/cureus.47885

**Published:** 2023-10-28

**Authors:** Sukjoo Cho, Alexandra Miller, Maua Mosha, Kevin O McNerney, Jonathan Metts

**Affiliations:** 1 Department of Pediatrics, University of South Florida Morsani College of Medicine, Tampa, USA; 2 Aflac Cancer and Blood Disorders Center, Children's Healthcare of Atlanta and Emory University, Atlanta, USA; 3 Data Coordinating Center for Pediatric Multicenter Studies, Institute for Clinical and Translational Research, Johns Hopkins All Children's Hospital, St. Petersburg, USA; 4 Data Coordinating Center for Pediatric Multicenter Studies, Institute for Clinical and Translational Research, Johns Hopkins All Children’s Hospital, St. Petersburg, USA; 5 Department of Pediatrics, Ann & Robert H. Lurie Children's Hospital of Chicago, Northwestern University Feinberg School of Medicine, Chicago, USA; 6 Cancer and Blood Disorders Institute, Johns Hopkins All Children's Hospital, St. Petersburg, USA; 7 Sarcoma Department, H. Lee Moffitt Cancer Center and Research Institute, Tampa, USA

**Keywords:** developmental therapeutics, cellular immunotherapy, cancer immunotherapy, pediatric hematology-oncology, clinical trials

## Abstract

Introduction: Cellular therapies are frequently studied in clinical trials for pediatric patients with malignant disease. Characteristics of ongoing and completed cellular therapy clinical trials in the U.S. involving children and adolescents have not previously been reported.

Methods: We searched ClinicalTrials.gov for clinical trials involving cellular therapies enrolling patients under 18 years of age in the U.S. Trials were initially stratified into child-only (maximum age of eligibility <18 years), child/adolescent and young adult (AYA) (maximum age of eligibility ≤21 years), and child/adult (maximum age of eligibility >21 years). Descriptive characteristics and trends over time were analyzed.

Results: We included 202 trials posted 2007-2022. Of the 202 trials, only three trials were child-only; thus, our subsequent analysis focused on comparing child/AYA (≤21 years) and child/adult trials (>21 years). One hundred sixty-nine (84%) enrolled both child and adult populations. The vast majority of trials were early phase (phase 1, 1/2, and 2, 198/202, 98%). Chimeric antigen receptor T cell therapies were most commonly studied (88/202, 44%), while natural-killer cell therapies were most common in child/AYA trials (42% vs. 16%). Most trials were single institution-only (130/202, 64%) and did not receive industry funding (163/202, 81%). Studies with industry funding were more likely to be multicenter (64% vs. 29%) and international (31% vs. 0.6%). Notably, no central nervous system tumor-specific trials had industry funding. There was no difference in therapy type based on funding source. Yearly new trial activations increased over the time period studied (p=0.01).

Conclusion: The frequency of cellular therapy trial activations enrolling child/AYA patients with cancer in the U.S. has increased over time. Most studies were phase 1 or 2, single institution-only, and not industry-supported. Future opportunities for cell therapy for pediatric cancer should include multi-institutional approaches.

## Introduction

Cellular therapy is the transfer of autologous or allogeneic cellular material for therapeutic purposes [1]. The study of non-hematopoietic stem cell transplant cellular therapy to treat oncologic diseases has accelerated dramatically in recent years and has seen some clinical success. Specifically, chimeric antigen receptor T-cells (CAR-T) have demonstrated striking responses in relapsed and refractory B-cell acute lymphoblastic leukemia (B-ALL) and B-cell lymphoma as well as multiple myeloma [2-6]. Additional successes have occurred in some solid tumors (ST) with adoptive transfer of tumor-infiltrating lymphocytes (TIL) and engineered T-cell receptors (TCR) as well as CAR-T [7-10]. Continued progress in immunology, genetic engineering, and synthetic biology have enhanced the potential of cellular therapy for oncologic and non-oncologic conditions [11]. As of April 2022, there were more than 2,700 active cellular therapy agents in development globally for the treatment of cancer, which was increased by 36% from a year prior [12].

The development of cellular therapy for pediatric malignancies has unique challenges. Above all, the scarcity of the diseases leading to recruitment difficulty is a known obstacle for clinical trials for rare pediatric diseases [13-15]. Along with the high cost required for research, manufacture, and administration of cell therapies, moreover, smaller support from industrial partners limits the discovery of novel cell therapeutics for children and adolescents [13,16,17]. Although not specific for pediatric indications, several barriers exist for use of cellular therapies, particularly for acute myeloid leukemia (AML), ST, and central nervous system (CNS) tumors, including imperfect target antigens [18-20]. Unlike CD19, which is typically uniformly expressed by malignant B-ALL and B-cell lymphoma cells, target antigens for myeloid leukemias and solid or CNS tumors may not have uniform expression. Additionally, target antigens may be shared with benign cells leading to “on-target off-tumor” toxicities [21]. 

While cellular therapy for pediatric cancer has been summarized in reviews, a formal analysis of ongoing and completed clinical trials of cellular therapy in pediatric cancer in the United States has not been investigated. Therefore, the primary objective of this study is to describe the characteristics of completed and ongoing trials using cell therapy for cancer that included children under the age of 18 years. The secondary objectives include comparing trials based on their overall age inclusion, the presence or absence of industry funding, and investigating temporal trends in these trials.

## Materials and methods

Data source and trial inclusion

ClinicalTrials.gov is a United States web-based registry with information on publicly- and privately-funded clinical studies, including clinical trials. Summaries of study protocols are presented including, but not limited to, diseases, interventions, study design, eligibility criteria, and study locations. Trial records were queried for those posted to ClinicalTrials.gov between September 27, 2007 to December 31, 2022. Of note, September 27, 2007 corresponds to the date of mandatory trial reporting in ClinicalTrials.gov by Section 801 of the Food and Drug Administration Amendments Act of 2007. These data were accessed on December 31, 2022. Trial posting dates may precede trial start dates, resulting in some trials with start dates after the date of data accession, including some trials with a listed start date in 2023. Trials starting in 2023 remained in the overall analysis, but were excluded in the temporal trend analysis. Exclusion criteria included studies enrolling only patients 18 years of age and older, studies not primarily studying an anti-cancer therapy, studies not utilizing a cellular therapy, and studies that were non-interventional or focused only on long-term follow-up from a prior trial. Additionally, studies only using hematopoietic stem cell transplant or modified stem cell products and not any additional form of cellular therapy were also excluded. Trials were filtered based on the condition search term “cancer” and other search terms to identify those trials involving cellular therapies (Table 1). Trials with a lower bound age of eligibility <18 years (using the automated age search filter for children on the ClinicalTrials.gov), with an interventional study type, and with one or more enrolling institutions in the United States were included. Trials were excluded if they were withdrawn before enrolling any patients. Two reviewers (K.M. and J.M.) performed independent manual reviews of all trials.

**Table 1:** Additional search terms to identify cellular therapy trials This is a list of search terms that we used on ClinicalTrials.gov to identify cellular therapy trials. CAR-T: chimeric antigen receptor T-cells, CIK: cytokine induced killer, APC: antigen presenting cell, ALT: adoptive lymphocyte transfer, TAA: tumor associated antigen, TSA: tumor specific antigen, NK: natural killer cells, NKT: natural killer T-cells

Search Terms
CAR-T
CIK
APC
ALT
TAA
TSA
NK
NKT
T cell receptor
chimeric
tumor specific antigen
cytokine induced killer
antigen presenting
adoptive transfer
cellular immunotherapy
tumor associated antigen
dendritic
autologous tumor
tumor vaccine

Study variables

The cancer type for each trial was categorized as ST, CNS, leukemia/lymphoma (LL), or enrolling multiple cancer types. The cellular therapies under investigation were classified as CAR-T, natural killer and natural killer T-cells (NK/NKT), TIL, TCR, dendritic cells, “other” T-cells (defined as any T-cell therapy not CAR-T, TIL, or TCR), autologous whole tumor cell infusions, or multiple cell types. We initially stratified patients into age categories defined as child-only (maximum age of eligibility <18 years), child/adolescent and young adult (AYA) (maximum age of eligibility ≤21 years), and child/adult (maximum age of eligibility >21 years). Sponsorship was categorized as industry, non-industry, or combined. Study sites were categorized as United States only or as international if at least one participating site was outside the United States. For number of sites, trials were categorized as single-institution or multiple-institution studies. For trials listed as “completed” on ClinicalTrials.gov at the time of data access, the trial duration was determined based on reported start date and primary completion date. For studies classified as “terminated” at the time of data access, the reason for termination was categorized as slow accrual, lack of funding, principal investigator/sponsor decision, or other. For geographic location, trials assigned geographic regions based on their study sites according to the four United States 2010 Census Regions: South, Northeast, Midwest, and West [22]. Trials with study locations in multiple geographic regions were assigned more than one region.

Statistical analysis

Characteristics of interest were stratified by the trial population to compare 1) child-only vs. child/AYA vs. child/adult trials and 2) trials that received any industry funding vs. no industry funding. Continuous variables were summarized as means with standard deviations or medians with interquartile ranges (IQR), and compared by T-Test or Mann-Whitney U test according to the sample distribution. Categorical variables were summarized as counts with percentages and compared using Chi-squared test or Fisher’s exact test as appropriate. The Mandal-Kenn test was used to investigate temporal trends in trial frequency. Temporal trends were analyzed from 2008 to 2022, where full-year data were available. All statistical analyses were two-sided, performed using R version 4.1.2 (2021-11-01; R Foundation for Statistical Computing, Vienna, Austria), and a p-value <0.05 was considered statistically significant.

## Results

Trial search and final inclusion

**Figure 1:**
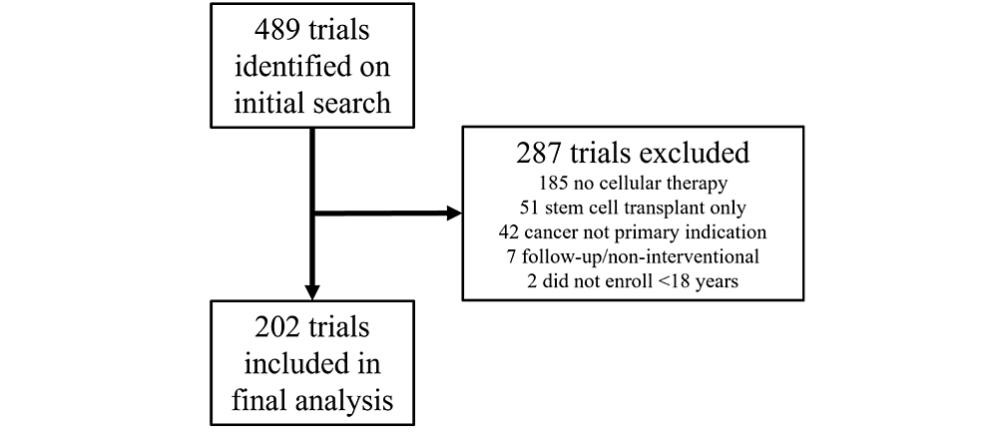
Flow diagram of trial inclusion/exclusion The flow diagram showing trials manually excluded, their reason for exclusion, and the final inclusion number after the initial ClinicalTrials.gov search

On initial search 489 trials were identified. On manual review, 287 trials were excluded based on the inclusion/exclusion criteria, leaving 202 trials for analysis (Figure 1). There were no final inclusion discrepancies between the two reviewers.

Overall characteristics of trials

Of 202 trials identified, only three trials exclusively enrolled patients under 18 years of age (child-only), including one trial of NK cells in high-grade glioma and two trials of T-cell-based therapy in neuroblastoma. Therefore, our subsequent analysis focused on comparing child/AYA (maximum age of eligibility ≤21 years) and child/adult groups (maximum age of eligibility >21 years). While 169 (84%) enrolled child/adult patients, 33 (16%) enrolled patients ≤21 years exclusively (child/AYA trials). Table 1 demonstrates the overall characteristics of these trials. The vast majority of trials were early phase (phase 1, 1/2, and 2, n=198, 98%) with only four phase 2/3 and 3 trials identified. T-cell-based therapies (CAR-T, TCR, TIL, and other T-cells) were the most common therapy type (n=137, 68%) with CAR-T the most prevalent (n=88, 44%). The majority of trials received no industry funding (n=163, 81%). Most studies were exclusive to the United States, with only 13 (6.4%) enrolling internationally. The majority of trials were single institution-only (n=130, 64%). Eighty-one trials (40%) were recruiting, and 40 trials (20%) were completed at the time of data accession. The median duration of completed trials was 5.3 years (range 1.1-9.8 years). Twenty-one trials (10%) were terminated, with the most common reason being slow accrual (n=10, 48% of terminated trials).

Table 2 also displays trial characteristics stratified by child/adult and child/AYA trials. Similar proportions of trials between these groups were seen across cancer type, trial phase, funding source, study site, reason for termination, and trial duration. The groups differed by therapy type (p=0.044); child/AYA trials had a higher proportion of NK/NKT cell-based therapies (42% vs 16%) and a lower proportion of CAR-T therapies (33% vs. 46%) than child/adult trials. Child/AYA trials were also notable for a higher percentage of single institution-only trials compared to child/adult trials (82% vs. 61%, p=0.022). Child/AYA and child/adult trials also differed in regard to study status (p=0.049). A higher proportion of child/AYA trials were listed as “not yet recruiting” (18% vs. 3.6%) and a lower proportion were “active, not recruiting” (9.1 vs. 24%) compared to child/adult trials.

**Table 2:** Characteristics of cellular therapy clinical trials enrolling children, stratified by age of eligibility AYA; adolescent and young adult, CNS; central nervous system, CAR-T; chimeric antigen receptor T cell, TCR; T cell receptor-engineered T cells, TIL; tumor-infiltrating lymphocytes, NK/NKT; natural killer/natural killer T cell, PI; primary investigator. ^1^N (%). ^2^Fisher's exact test; Pearson's Chi-squared test; Mann-Whitney U test.

Category	Variable	Overall, N=202^1^	Child/Adult, N=169^1^	Child/AYA, N=33^1^	P-value^2^
Cancer Type	Leukemia/Lymphoma	110 (54%)	94 (56%)	16 (48%)	0.8
	CNS Tumors	24 (12%)	19 (11%)	5 (15%)	
	Solid Tumor	57 (28%)	46 (27%)	11 (33%)	
	Multiple	11 (5.4%)	10 (5.9%)	1 (3.0%)	
Trial Phase	Phase 1	123 (61%)	104 (62%)	19 (58%)	0.2
	Phase 1/2 or 2	75 (37%)	63 (37%)	12 (36%)	
	Phase 2/3 or 3	4 (2.0%)	2 (1.2%)	2 (6.1%)	
Therapy Type	CAR-T	88 (44%)	77 (46%)	11 (33%)	0.044
	Other T Cell	33 (16%)	29 (17%)	4 (12%)	
	TCR	11 (5.4%)	8 (4.7%)	3 (9.1%)	
	TIL	5 (2.5%)	5 (3.0%)	0 (0%)	
	NK/NKT	41 (20%)	27 (16%)	14 (42%)	
	Dendritic Cell	10 (5.0%)	10 (5.9%)	0 (0%)	
	Autologous Tumor Cells	6 (3.0%)	6 (3.6%)	0 (0%)	
	Multiple	8 (4.0%)	7 (4.1%)	1 (3.0%)	
Funding Source	Industry	23 (11%)	20 (12%)	3 (9.1%)	>0.9
	Combined	16 (7.9%)	14 (8.3%)	2 (6.1%)	
	Non-Industry	163 (81%)	135 (80%)	28 (85%)	
Study Site	United States	189 (94%)	159 (94%)	30 (91%)	0.4
	International	13 (6.4%)	10 (5.9%)	3 (9.1%)	
Number of Sites	Single	130 (64%)	103 (61%)	27 (82%)	0.022
	Multiple	72 (36%)	66 (39%)	6 (18%)	
Study Status	Recruiting	81 (40%)	69 (41%)	12 (36%)	0.049
	Enrolling by Invitation	1 (0.5%)	1 (0.6%)	0 (0%)	
	Not Yet Recruiting	12 (5.9%)	6 (3.6%)	6 (18%)	
	Completed	40 (20%)	31 (18%)	9 (27%)	
	Active, Not Recruiting	43 (21%)	40 (24%)	3 (9.1%)	
	Terminated	21 (10%)	18 (11%)	3 (9.1%)	
	Suspended	1 (0.5%)	1 (0.6%)	0 (0%)	
	Unknown Status	3 (1.5%)	3 (1.8%)	0 (0%)	
Reason for Termination	N	21	18	3	0.8
	Slow Accrual	10 (48%)	9 (50%)	1 (33%)	
	Lack of Funding	2 (9.5%)	2 (11%)	0 (0%)	
	PI/Sponsor Decision	3 (14%)	2 (11%)	1 (33%)	
	Other	6 (29%)	5 (28%)	1 (33%)	
Duration of Trial (years, completed only)	N	40	31	9	>0.9
	Median (IQR)	5.30 (3.10, 7.32)	5.20 (3.15, 7.00)	5.40 (3.20, 8.20)	
	Range	1.10, 9.80	1.10, 9.80	2.20, 8.60	

Table 3 displays characteristics stratified by source of funding (any industry funding vs. no industry funding). These two groups differed by tumor type (p=0.014); notably, no CNS tumor-specific trials received any industry funding. The groups also differed by trial phase (p<0.001), with a higher percentage of trials without industry funding being phase 1 (67% vs. 36%). Trials receiving industry funding were proportionately more international (31% vs. 0.6%, p<0.001) and multi-site (64% vs. 47%, p<0.001). Similar proportions were seen across therapy type, age of eligibility, study status, reason for termination, and trial duration.

**Table 3:** Characteristics of cellular therapy clinical trials enrolling children, stratified by funding source CNS; central nervous system, CAR-T; chimeric antigen receptor T cell, TCR; T cell receptor-engineered T cells, TIL; tumor-infiltrating lymphocytes, NK/NKT; natural killer/natural killer T cell, AYA; adolescent and young adult, PI; primary investigator. ^1^N (%). ^2^Fisher's exact test; Pearson's Chi-squared test; Mann-Whitney U test.

Category	Variable	Overall, N=202^1^	Non-Industry, N=163^1^	Any Industry Funding, N=39^1^	P-value^2^
Tumor Type	Leukemia/Lymphoma	110 (54%)	89 (55%)	21 (54%)	0.014
	CNS Tumors	24 (12%)	24 (15%)	0 (0%)	
	Solid Tumor	57 (28%)	41 (25%)	16 (41%)	
	Multiple	11 (5.4%)	9 (5.5%)	2 (5.1%)	
Trial Phase	Phase 1	123 (61%)	109 (67%)	14 (36%)	<0.001
	Phase 1/2 or 2	75 (37%)	54 (33%)	21 (54%)	
	Phase 2/3 or 3	4 (2.0%)	0 (0%)	4 (10%)	
Therapy Type	CAR-T	88 (44%)	73 (45%)	15 (38%)	0.12
	Other T Cell	33 (16%)	27 (17%)	6 (15%)	
	TCR	11 (5.4%)	6 (3.7%)	5 (13%)	
	TIL	5 (2.5%)	3 (1.8%)	2 (5.1%)	
	NK/NKT	41 (20%)	35 (21%)	6 (15%)	
	Dendritic Cell	10 (5.0%)	9 (5.5%)	1 (2.6%)	
	Tumor Cells	6 (3.0%)	3 (1.8%)	3 (7.7%)	
	Multiple	8 (4.0%)	7 (4.3%)	1 (2.6%)	
Age of Eligibility	Child/Adult	169 (84%)	135 (83%)	34 (87%)	0.5
	Child/AYA	33 (16%)	28 (17%)	5 (13%)	
Study Site	US	189 (94%)	162 (99%)	27 (69%)	<0.001
	International	13 (6.4%)	1 (0.6%)	12 (31%)	
Number of Sites	Single	130 (64%)	116 (71%)	14 (36%)	<0.001
	Multiple	72 (36%)	47 (29%)	25 (64%)	
Study Status	Recruiting	81 (40%)	65 (40%)	16 (41%)	>0.9
	Enrolling by Invitation	1 (0.5%)	1 (0.6%)	0 (0%)	
	Not Yet Recruiting	12 (5.9%)	10 (6.1%)	2 (5.1%)	
	Completed	40 (20%)	33 (20%)	7 (18%)	
	Active, Not Recruiting	43 (21%)	34 (21%)	9 (23%)	
	Terminated	21 (10%)	16 (9.8%)	5 (13%)	
	Suspended	1 (0.5%)	1 (0.6%)	0 (0%)	
	Unknown Status	3 (1.5%)	3 (1.8%)	0 (0%)	
Reason for Termination	N	21	16	5	0.3
	Slow Accrual	10 (48%)	8 (50%)	2 (40%)	
	Lack of Funding	2 (9.5%)	2 (12%)	0 (0%)	
	PI/Sponsor Decision	3 (14%)	1 (6.2%)	2 (40%)	
	Other	6 (29%)	5 (31%)	1 (20%)	
Duration of Trial (years, completed only)	N	40	33	7	0.4
	Median (IQR)	5.30 (3.10, 7.32)	5.20 (3.20, 6.70)	6.70 (3.35, 8.10)	
	Range	1.10, 9.80	1.10, 9.80	2.20, 9.00	

**Figure 2:**
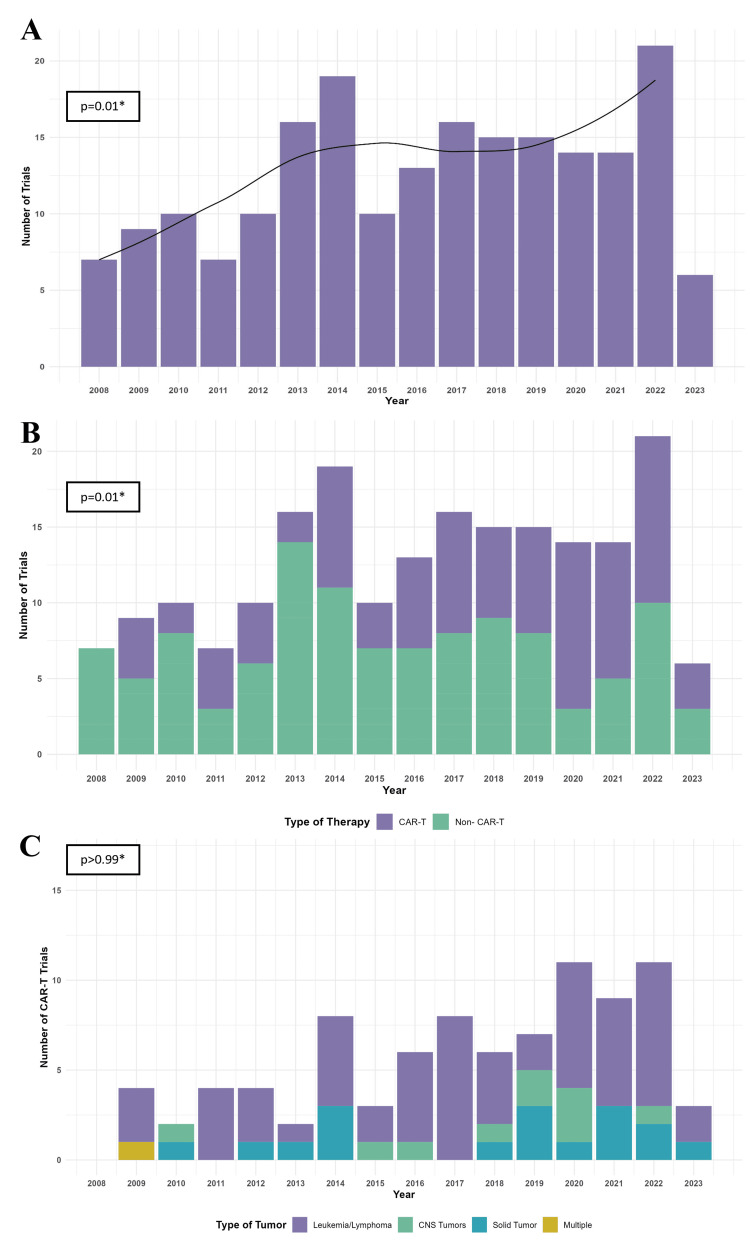
Trends in cellular therapy trials 2008-2022 A) Included trials organized by year of their activation. No trials had a start date in 2007, and six trials included a start date in 2023. The Mann-Kendall test for trend was performed for years 2008-2022. B) Trials organized by year of activation and classified as CAR-T therapy or Non-CAR-T therapy. The Mann-Kendall test for trend was performed for years 2008-2022. C) CAR-T therapy trials organized by year and classified by disease type treated. The Mann-Kendall test for trend was performed for years 2008-2022.

**Figure 3:**
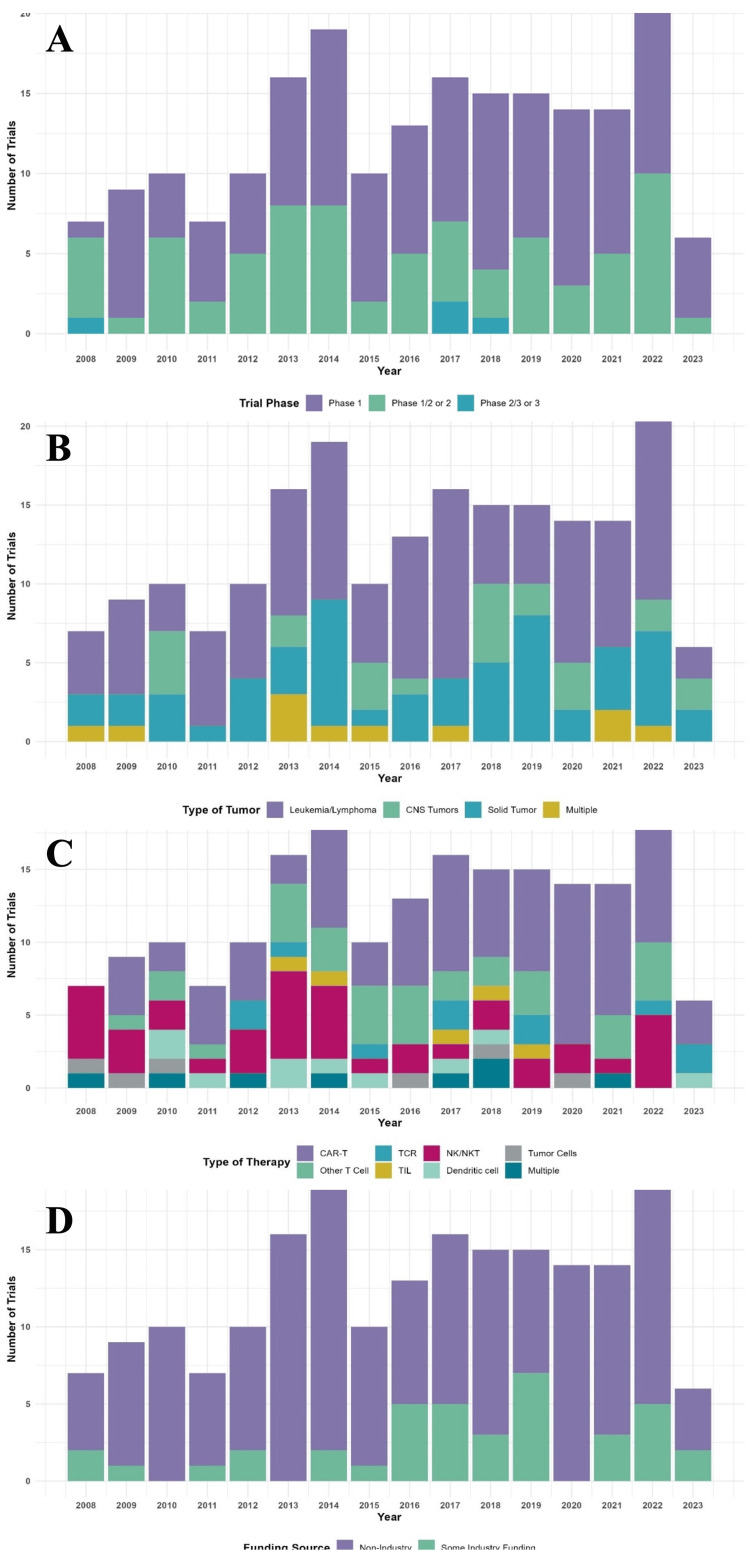
Temporal trends in cellular therapy trials by trial phase, cancer type, individual cell therapy types, and funding sources A) Clinical trials by year of activation and phase. B) Clinical trials by year of activation and cancer type. C) Clinical trials by year of activation and therapy type. D) Clinical trials by year of activation and funding source.

Trial activations by year of listed start date are displayed in Figure 2A. There was an increase in the frequency of new cellular therapy trial activations from the first reported trial start dates in 2008 through 2022 (p=0.01). For years with complete data available (2008-2022), there was a median of 14 (range 7 to 21) cellular therapy trials activated each year, peaking with 21 trial activations in 2022. For cell therapy type, notably CAR-T therapy trials increased in comparison to non-CAR-T therapy trials over the study period (Figure 2B, p=0.01). Within CAR-T trials, a specific trend in proportions of disease types was not noted (Figure 2C, p>0.99). Trials were then investigated for temporal trends based on trial phase, cancer type, individual cell therapy types, and funding sources (Figure 3). No temporal trends were found within these categories. Notably, there was not an increase in phase 2/3 or phase 3 trial activations over time, with the most recent phase 2/3 or phase 3 trial activating in 2018.

**Figure 4:**
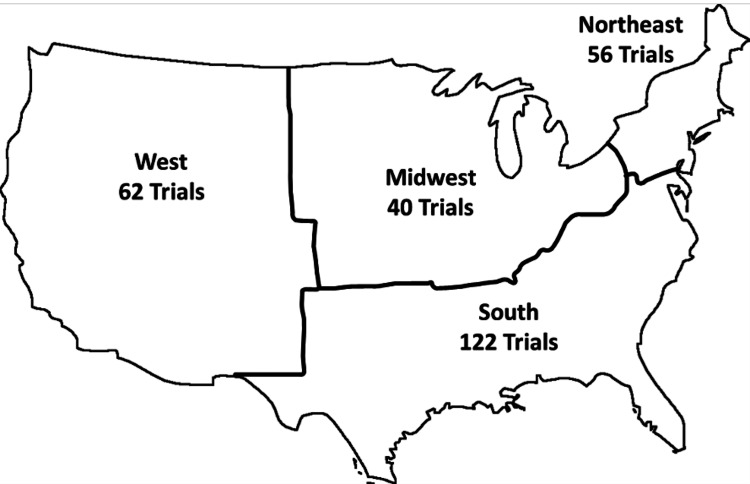
Clinical trials by region Schematic of the United States divided into 2010 census-designated geographic regions, with the number of trials enrolling during the study period shown in each region. Some trials enrolled in more than one region, thus the sum of the trials on this graph is higher than the number of trials studied.

In regards to regional availability, given the proportion of trials that were single institution-only, most trials were confined to one geographic region (n=166, 82%), with 36 (18%) of trials spanning more than one geographic region. The South region had the largest number of available cellular therapy trials during the study period with 122, followed by the West with 62 trials, the Northeast with 56 trials, and the Midwest with 40 trials (Figure 4).

## Discussion

We conducted an analysis of 202 clinical trials for cellular therapy for child and adolescent patients with cancer from 2007 to 2022, using a publicly accessible registry in the United States. Our findings describe the landscape of cell therapy trials for children and adolescents with cancer, thereby providing novel insights into the design of future clinical trials. Very few trials (n=3) enrolled exclusively children <18 years. As many pediatric centers care for patients up to 21 or occasionally higher, however, our analysis of child/AYA trials enrolling up to 21 years of age remains relevant for pediatric oncologists. Most identified trials were early phase (phase 1, 1/2, and 2), single institution, and only available at U.S. centers. Only a fraction of trials exclusively enrolled child/AYA patients, while the others were open to both child and adult patients. LL was the most common disease type for trials, and T-cell-based therapies, particularly CAR-T, were the most investigated cell therapy type, although NK/NKT-cell-based therapies were more commonly studied among child/AYA trials. Most trials were not supported by industry; of note, there was no CNS tumor study receiving any industrial funding. We found an overall increase in new trial activation in the years studied.

The prevalence of early-phase trials in our analysis is likely multifactorial. First, the finding probably reflects constant expansion and evolution of the cellular therapy field and the general abundance of early-phase trials representing immediate translation of preclinical work. In fact, according to a recent report, phase 1 and 2 studies accounted for 88% of active trials available at the ClinicalTrials.gov [12]. Second, the rarity of pediatric cancer could contribute to the proportion of early-phase studies. It is known that rare disease trials are more likely to be early phase compared to non-rare disease trials [23]. This might be due to small patient populations and fragmented drug development for the rare diseases [24]. Third, the timeline for development of new drugs for pediatric cancer is generally longer than adult counterpart. First-in-child trials are usually initiated many years after first-in-human trials [15]. In addition, industry rarely pursues first-in-child studies because of small incentives; moreover, there were several phase 2 trials with positive results that have not progressed to phase 3 due to lack of industrial interest [16]. Varying regulatory standards between adult and pediatric studies is another hurdle that delays the drug development process [15,16,25].

The predominance of single institution-only trials elicits two different perspectives on future directions. In the sense that the cost and regulatory burdens of cellular therapy development can be minimized, the single-institution approach has its own value. Considering the rarity of cell therapy indications (e.g., relapsed/refractory cancers with dismal prognosis) and resultant slow accrual, however, multicenter approaches would make cell therapy trial opportunities available to a wider population and potentially facilitate recruitment. in addition, expecting that cellular therapeutics will be more widely used and eventually incorporated into up-front treatment regimens, concerted efforts toward multisite trials may better position the field for more broad use of cell therapies in the future. Taken together, we may consider a single-center trial in earlier phases to streamline the trial design and then a multicenter trial in later phases to maximize generalizability [26].

After the initial success in B-ALL, CAR-T has led in number of clinical trials and increased in proportion over time. Although sharp and steady growth of CAR-T studies led the expansion of the entire cell therapy pipeline, evidence of CAR-T efficacy has lagged in myeloid leukemias and solid tumors [12]. One of the major obstacles to advancing indications of CAR-T is imperfect targets. Many trials investigating CAR-T for ST and CNS tumors focused on cell surface antigens that are expressed at high levels on malignant cells but scarcely discovered in normal tissues, including B7-H3/CD276 and GD2 [10,27-31]. Such molecules are not typically cancer specific but found on various tumors. Given that even the most common pediatric ST are extremely rare, identifying one target molecule shared by many different diseases would make the biggest impact [32]. It is promising that preliminary reports of recent trials reported feasibility and safety of the drugs, including early evidence of activity signal in diseases with dismal prognosis such as diffuse midline gliomas and relapsed and refractory high-risk neuroblastoma [10,31,33,34]. When it comes to AML, many myeloid surface antigens are not leukemia specific and often co-expressed on normal hematopoietic stem/progenitor cells [35]. Therefore, CAR-T targeting such antigens may induce prolonged myeloablation with resultant neutropenic infections and bleeding complications, and require subsequent hematopoietic stem cell transplantation. Efforts to discover effective and safe CAR-T targeting novel AML antigens are ongoing [36].

Of note, NK/NKT therapies were the most commonly investigated cellular therapy in child/AYA clinical trials. NK/NKT therapies may play an important role in pediatric cancer treatment due to unique reasons. First, many pediatric malignancies require conventional chemotherapy which may result in profound immunodeficiency, particularly secondary to depletion of T-cells [37-40]. Accumulating evidence showed that such T-cell deficits may interfere with long-term remission and impair the potential of T-cell-based therapy [41-43]. On the other hand, the antitumor immunity of NK cells is relatively preserved after chemoradiation [39,44]. As compared to CAR-T, NK/NKT cell therapy has superior safety and retained efficacy in the allogenic setting, offering a potential for the off-the-shelf product [45,46].

There are several limitations to our study. First, the Public Health Service Act of the United States does not consider phase 1 trials to be “applicable clinical trials” for mandated registration on ClinicalTrials.gov [47]. Therefore, our analysis does not account for phase 1 trials not available on ClinicalTrials.gov. Second, while we diligently selected our search terms to comprehensively identify all cellular therapy trials enrolling patients <18 years of age, it is possible that there are posted trials that were not identified by our search strategy and thus not represented here. Third, our analysis does not address the actual number of pediatric patients who were enrolled in these cellular therapy trials. Finally, molecular targets of cell therapies were not captured in our analysis, although such information would be interesting to review given that many target antigens are shared among different types of pediatric cancer.

## Conclusions

In conclusion, our report provides novel insights into the current landscape of pediatric cancer cell therapy trials. Overall up-trending new and early phase trials as well as investigation of various approaches including CAR-T, TCR, and NK/NKT are promising. Considering that cellular therapy is expected to play a pivotal role in management of various pediatric cancers, robust investigation must be continued. Particularly, future opportunities should include multi-institutional approaches given the rarity of childhood and adolescent cancers. Our analysis will serve as a benchmark for future analyses on the development of cellular therapy for children and adolescents.
